# Cystal structre of 5-hy­droxy-2-nitro­benzaldehyde

**DOI:** 10.1107/S205698901500701X

**Published:** 2015-04-22

**Authors:** Huma Bano, Sammer Yousuf

**Affiliations:** a H.E.J. Research Institute of Chemistry, International Center for Chemical and Biological Sciences, University of Karachi, Karachi 75270, Pakistan

**Keywords:** crystal structure, nitro-substituted aromatics, O—H⋯O hydrogen bonds, C—H⋯O hydrogen bonds

## Abstract

In the title compound, C_7_H_5_NO_4_, the nitro group and the aldehyde group are inclined to the benzene ring by 16.6 (3) and 15.6 (3)°, respectively. In the crystal, mol­ecules are linked *via* O—H⋯O hydrogen bonds, forming chains along [100]. The chains are linked by C—H⋯O hydrogen bonds, forming a three-dimensional structure.

## Related literature   

For literature on nitro-substituted aromatic compounds and their various properties, see: Yan *et al.* (2006 ▸); Soojhawon *et al.* (2005 ▸). For crystal structures of related compounds, see: Tang *et al.* (2010 ▸); Tanak *et al.* (2009 ▸); Singh *et al.* (2009 ▸).
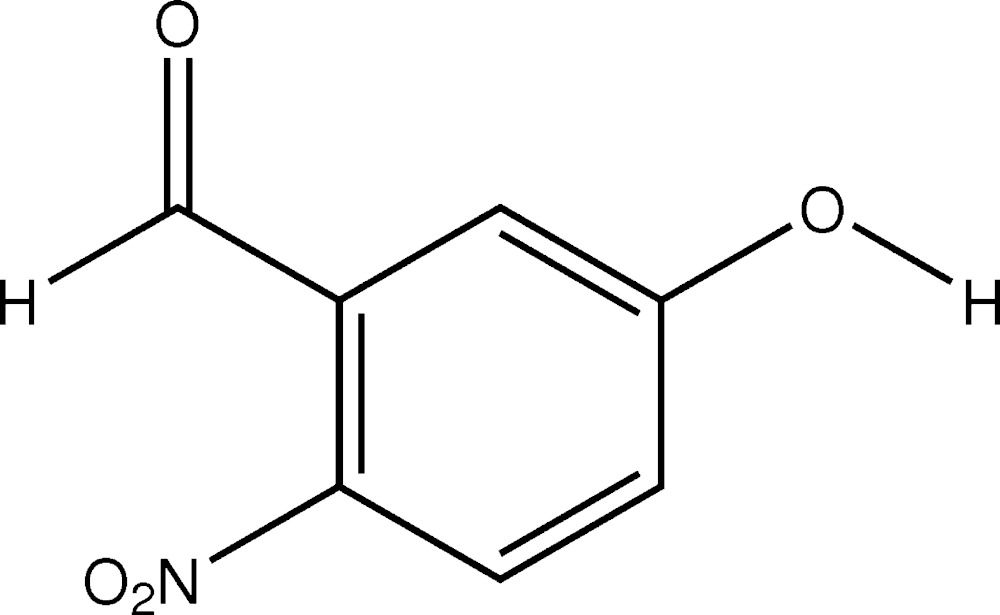


## Experimental   

### Crystal data  

C_7_H_5_NO_4_
*M* *_r_* = 167.12
Monoclinic, 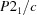

*a* = 9.6648 (18) Å
*b* = 5.0917 (10) Å
*c* = 14.920 (3) Å
β = 106.159 (4)°
*V* = 705.2 (2) Å^3^
*Z* = 4
Mo *K*α radiation
μ = 0.13 mm^−1^
*T* = 273 K
0.48 × 0.32 × 0.15 mm

### Data collection  

Bruker SMART APEX CCD area-detector diffractometer
Absorption correction: multi-scan (*SADABS*; Bruker, 2000 ▸) *T*_min_ = 0.939, *T*_max_ = 0.980
3884 measured reflections
1312 independent reflections
974 reflections with *I* > 2σ(*I*)
*R*_int_ = 0.024

### Refinement  

*R*[*F* ^2^ > 2σ(*F* ^2^)] = 0.046
*wR*(*F* ^2^) = 0.116
*S* = 1.04
1312 reflections
113 parameters
H atoms treated by a mixture of independent and constrained refinement
Δρ_max_ = 0.19 e Å^−3^
Δρ_min_ = −0.16 e Å^−3^

### 

Data collection: *SMART* (Bruker, 2000 ▸); cell refinement: *SAINT* (Bruker, 2000 ▸); data reduction: *SAINT*; program(s) used to solve structure: *SHELXS97* (Sheldrick, 2008 ▸); program(s) used to refine structure: *SHELXL97* (Sheldrick, 2008 ▸); molecular graphics: *SHELXTL* (Sheldrick, 2008 ▸) and *Mercury* (Macrae *et al.*, 2008 ▸); software used to prepare material for publication: *SHELXTL* (Sheldrick, 2008 ▸) and *PLATON* (Spek, 2009 ▸).

## Supplementary Material

Crystal structure: contains datablock(s) global, I. DOI: 10.1107/S205698901500701X/su5113sup1.cif

Structure factors: contains datablock(s) I. DOI: 10.1107/S205698901500701X/su5113Isup2.hkl

Supporting information file. DOI: 10.1107/S205698901500701X/su5113Isup3.cml

**Fig. 1.**
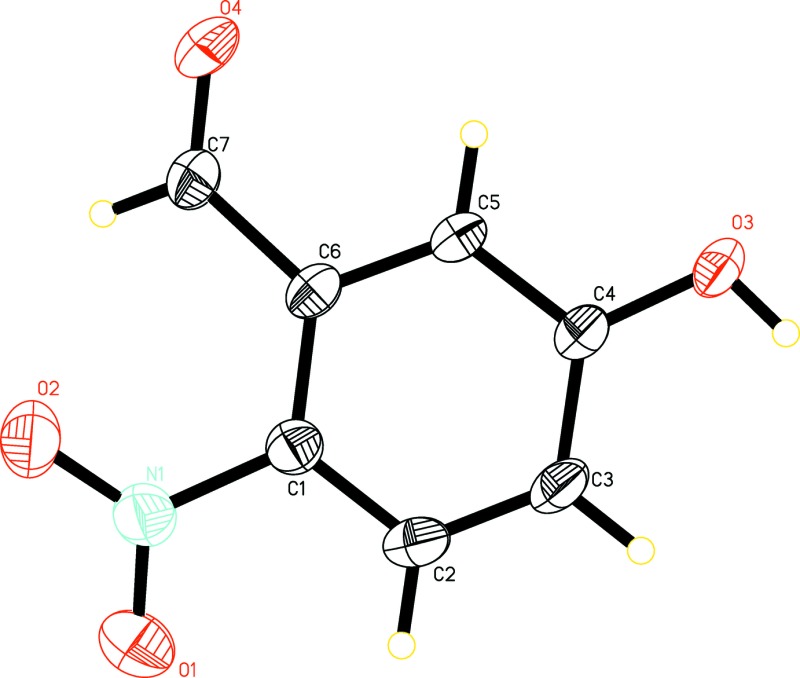
The molecular structure of title compound, with atom labelling. Displacement ellipsoids are drawn at 30% probability level.

**Fig. 2.**
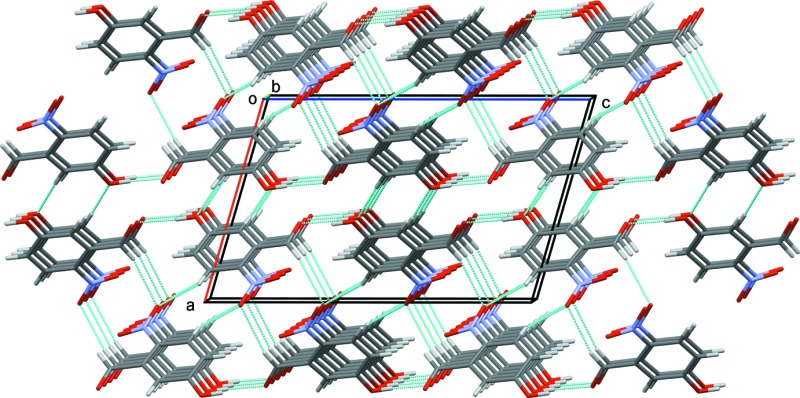
The crystal packing of title compound, viewed along the b axis. Hydrogen bonds are shown as dashed lines (see Table 1 for details).

. DOI: 10.1107/S205698901500701X/su5113fig1.tif
The mol­ecular structure of title compound, with atom labelling. Displacement ellipsoids are drawn at 30% probability level.

b . DOI: 10.1107/S205698901500701X/su5113fig2.tif
The crystal packing of title compound, viewed along the *b* axis. Hydrogen bonds are shown as dashed lines (see Table 1 for details).

CCDC reference: 1058381

Additional supporting information:  crystallographic information; 3D view; checkCIF report

## Figures and Tables

**Table 1:** Hydrogen-bond geometry (, )

*D*H*A*	*D*H	H*A*	*D* *A*	*D*H*A*
O3H3*B*O4^i^	0.88(3)	1.82(3)	2.699(2)	174(3)
C2H2*A*O1^ii^	0.93	2.48	3.364(3)	158
C5H5*A*O3^iii^	0.93	2.45	3.379(3)	173
C7H7*A*O1^iv^	0.93	2.49	3.264(3)	140

Symmetry codes: (i) 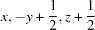; (ii) 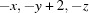; (iii) 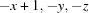; (iv) 
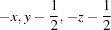
.

## supplementary crystallographic information

### S1. Synthesis and crystallization

Colourless crystals of the title compound [Fluka; HPLC grade] were obtained by slow evaporation of a solution in methanol.

### S2. Refinement

Crystal data, data collection and structure refinement details are summarized in Table 2. The hydroxyl H atom was located in a difference Fourier map and freely refined. The C-bound H atoms were positioned geometrically and constrained to ride on their parent atoms: C—H = 0.93 Å, with *U*_iso_(H) = 1.2*U*_eq_(C).

### S3. Comment

Nitro substituted aromatic compounds are well known inter­mediates in various organic reactions, responsible for synthesis of pesticides, explosive materials and other bioactive phenyl derivatives (Yan *et al.*, 2006). The nitro substituted aromatic compounds are also known to be widely distributed as pollutant in air and water reservoirs (Yan *et al.*, 2006; Soojhawon *et al.*, 2005). The title compound is a commercially available benzaldehyde derivative, composed of a planar hy­droxy substituted nitro­benzaldehyde ring. The compound was crystal out as a part of our ongoing research project involving to crystallize and evaluate biological activities of commercially available molecular libraries.

The molecular structure of the title compound is illustrated in Fig. 1. Structurally it is a positional isomer of the previously reported 2-hy­droxy-5-nitro­benzaldehyde with the difference that the positions of the hy­droxy and nitro substituents are inter­changed (Tanak *et al.*, 2009). The nitro group (N1/O1/O2) and the aldehyde group (C6/C1/O4) are inclined to the benzene ring (C1—C6) by 16.6 (3) and 15.6 (3) °, respectively.

In the crystal, molecules are linked by O—H···O hydrogen bonds forming zigzag chains along [100]. The chains are linked via C—H···O hydrogen bonds forming a three dimensional structure (Table 2 and Fig. 2).

### Crystal data

**Table:**

C_7_H_5_NO_4_	*F*(000) = 344
*M**_r_* = 167.12	*D*_x_ = 1.574 Mg m^−^^3^
Monoclinic, *P*2_1_/*c*	Mo *K*α radiation, λ = 0.71073 Å
Hall symbol: P 2ybc	Cell parameters from 924 reflections
*a* = 9.6648 (18) Å	θ = 2.8–23.7°
*b* = 5.0917 (10) Å	µ = 0.13 mm^−^^1^
*c* = 14.920 (3) Å	*T* = 273 K
β = 106.159 (4)°	Block, colourles
*V* = 705.2 (2) Å^3^	0.48 × 0.32 × 0.15 mm
*Z* = 4	

### Data collection

**Table:**

Bruker SMART APEX CCD area-detector diffractometer	1312 independent reflections
Radiation source: fine-focus sealed tube	974 reflections with *I* > 2σ(*I*)
Graphite monochromator	*R*_int_ = 0.024
ω scan	θ_max_ = 25.5°, θ_min_ = 2.2°
Absorption correction: multi-scan (*SADABS*; Bruker, 2000)	*h* = −11→11
*T*_min_ = 0.939, *T*_max_ = 0.980	*k* = −5→6
3884 measured reflections	*l* = −18→16

### Refinement

**Table:**

Refinement on *F*^2^	Primary atom site location: structure-invariant direct methods
Least-squares matrix: full	Secondary atom site location: difference Fourier map
*R*[*F*^2^ > 2σ(*F*^2^)] = 0.046	Hydrogen site location: inferred from neighbouring sites
*wR*(*F*^2^) = 0.116	H atoms treated by a mixture of independent and constrained refinement
*S* = 1.04	*w* = 1/[σ^2^(*F*_o_^2^) + (0.0518*P*)^2^ + 0.1669*P*] where *P* = (*F*_o_^2^ + 2*F*_c_^2^)/3
1312 reflections	(Δ/σ)_max_ < 0.001
113 parameters	Δρ_max_ = 0.19 e Å^−^^3^
0 restraints	Δρ_min_ = −0.16 e Å^−^^3^

### Special details

**Table:**

Geometry. All e.s.d.'s (except the e.s.d. in the dihedral angle between two l.s. planes) are estimated using the full covariance matrix. The cell e.s.d.'s are taken into account individually in the estimation of e.s.d.'s in distances, angles and torsion angles; correlations between e.s.d.'s in cell parameters are only used when they are defined by crystal symmetry. An approximate (isotropic) treatment of cell e.s.d.'s is used for estimating e.s.d.'s involving l.s. planes.
Refinement. Refinement of *F*^2^ against ALL reflections. The weighted *R*-factor *wR* and goodness of fit *S* are based on *F*^2^, conventional *R*-factors *R* are based on *F*, with *F* set to zero for negative *F*^2^. The threshold expression of *F*^2^ > σ(*F*^2^) is used only for calculating *R*-factors(gt) *etc*. and is not relevant to the choice of reflections for refinement. *R*-factors based on *F*^2^ are statistically about twice as large as those based on *F*, and *R*- factors based on ALL data will be even larger.

### Fractional atomic coordinates and isotropic or equivalent isotropic displacement parameters (Å^2^)

**Table:**

	*x*	*y*	*z*	*U*_iso_*/*U*_eq_	
O1	0.0034 (2)	0.9749 (4)	−0.12613 (15)	0.0961 (7)	
O2	0.1401 (2)	0.9319 (3)	−0.21588 (12)	0.0763 (6)	
O3	0.42366 (18)	0.1329 (3)	0.09957 (10)	0.0571 (5)	
O4	0.39446 (19)	0.3765 (4)	−0.22575 (10)	0.0671 (5)	
N1	0.1039 (2)	0.8710 (4)	−0.14662 (14)	0.0577 (5)	
C1	0.1847 (2)	0.6689 (4)	−0.08470 (13)	0.0440 (5)	
C2	0.1690 (2)	0.6549 (4)	0.00439 (15)	0.0524 (6)	
H2A	0.1050	0.7672	0.0218	0.063*	
C3	0.2468 (2)	0.4774 (4)	0.06721 (14)	0.0504 (6)	
H3A	0.2356	0.4691	0.1271	0.061*	
C4	0.3419 (2)	0.3104 (4)	0.04173 (12)	0.0421 (5)	
C5	0.3562 (2)	0.3232 (4)	−0.04816 (12)	0.0420 (5)	
H5A	0.4192	0.2086	−0.0654	0.050*	
C6	0.2793 (2)	0.5012 (4)	−0.11264 (12)	0.0408 (5)	
C7	0.2988 (3)	0.4856 (4)	−0.20759 (14)	0.0543 (6)	
H7A	0.2307	0.5677	−0.2560	0.065*	
H3B	0.411 (3)	0.140 (5)	0.156 (2)	0.078 (8)*	

### Atomic displacement parameters (Å^2^)

**Table:**

	*U*^11^	*U*^22^	*U*^33^	*U*^12^	*U*^13^	*U*^23^
O1	0.0934 (14)	0.1045 (16)	0.0934 (14)	0.0513 (13)	0.0311 (12)	0.0152 (12)
O2	0.1128 (15)	0.0612 (11)	0.0566 (10)	0.0066 (10)	0.0265 (10)	0.0107 (8)
O3	0.0801 (11)	0.0627 (10)	0.0343 (8)	0.0137 (8)	0.0256 (8)	0.0081 (7)
O4	0.0813 (11)	0.0887 (13)	0.0398 (8)	0.0127 (10)	0.0307 (8)	−0.0016 (8)
N1	0.0660 (13)	0.0515 (12)	0.0528 (11)	0.0033 (10)	0.0117 (10)	−0.0038 (9)
C1	0.0457 (11)	0.0436 (11)	0.0427 (11)	−0.0009 (9)	0.0123 (9)	−0.0025 (9)
C2	0.0578 (13)	0.0528 (13)	0.0542 (13)	0.0027 (11)	0.0280 (11)	−0.0087 (10)
C3	0.0650 (14)	0.0565 (13)	0.0377 (11)	−0.0020 (11)	0.0273 (11)	−0.0030 (10)
C4	0.0518 (12)	0.0423 (11)	0.0354 (10)	−0.0046 (9)	0.0175 (9)	−0.0022 (9)
C5	0.0479 (11)	0.0477 (12)	0.0342 (10)	−0.0005 (9)	0.0177 (9)	−0.0063 (9)
C6	0.0439 (11)	0.0466 (11)	0.0333 (10)	−0.0081 (9)	0.0129 (9)	−0.0064 (8)
C7	0.0685 (15)	0.0608 (14)	0.0337 (11)	0.0090 (12)	0.0144 (11)	0.0026 (10)

### Geometric parameters (Å, º)

**Table:**

O1—N1	1.218 (2)	C2—H2A	0.9300
O2—N1	1.219 (3)	C3—C4	1.381 (3)
O3—C4	1.344 (2)	C3—H3A	0.9300
O3—H3B	0.88 (3)	C4—C5	1.387 (3)
O4—C7	1.173 (2)	C5—C6	1.379 (3)
N1—C1	1.457 (3)	C5—H5A	0.9300
C1—C2	1.381 (3)	C6—C7	1.482 (3)
C1—C6	1.397 (3)	C7—H7A	0.9300
C2—C3	1.367 (3)		
			
C4—O3—H3B	111.7 (17)	O3—C4—C3	123.70 (17)
O1—N1—O2	122.6 (2)	O3—C4—C5	116.94 (18)
O1—N1—C1	118.2 (2)	C3—C4—C5	119.36 (18)
O2—N1—C1	119.2 (2)	C6—C5—C4	121.73 (18)
C2—C1—C6	120.82 (19)	C6—C5—H5A	119.1
C2—C1—N1	117.63 (19)	C4—C5—H5A	119.1
C6—C1—N1	121.50 (18)	C5—C6—C1	117.64 (17)
C3—C2—C1	120.47 (19)	C5—C6—C7	116.39 (18)
C3—C2—H2A	119.8	C1—C6—C7	125.87 (19)
C1—C2—H2A	119.8	O4—C7—C6	124.3 (2)
C2—C3—C4	119.97 (18)	O4—C7—H7A	117.8
C2—C3—H3A	120.0	C6—C7—H7A	117.8
C4—C3—H3A	120.0		
			
O1—N1—C1—C2	−16.9 (3)	C3—C4—C5—C6	1.0 (3)
O2—N1—C1—C2	162.27 (19)	C4—C5—C6—C1	−0.5 (3)
O1—N1—C1—C6	165.7 (2)	C4—C5—C6—C7	−176.99 (18)
O2—N1—C1—C6	−15.2 (3)	C2—C1—C6—C5	−0.2 (3)
C6—C1—C2—C3	0.4 (3)	N1—C1—C6—C5	177.18 (18)
N1—C1—C2—C3	−177.03 (19)	C2—C1—C6—C7	175.9 (2)
C1—C2—C3—C4	0.0 (3)	N1—C1—C6—C7	−6.7 (3)
C2—C3—C4—O3	179.1 (2)	C5—C6—C7—O4	−17.1 (3)
C2—C3—C4—C5	−0.7 (3)	C1—C6—C7—O4	166.8 (2)
O3—C4—C5—C6	−178.86 (18)		

### Hydrogen-bond geometry (Å, º)

**Table:**

*D*—H···*A*	*D*—H	H···*A*	*D*···*A*	*D*—H···*A*
O3—H3*B*···O4^i^	0.88 (3)	1.82 (3)	2.699 (2)	174 (3)
C2—H2*A*···O1^ii^	0.93	2.48	3.364 (3)	158
C5—H5*A*···O3^iii^	0.93	2.45	3.379 (3)	173
C7—H7*A*···O1^iv^	0.93	2.49	3.264 (3)	140

Symmetry codes: (i) *x*, −*y*+1/2, *z*+1/2; (ii) −*x*, −*y*+2, −*z*; (iii) −*x*+1, −*y*, −*z*; (iv) −*x*, *y*−1/2, −*z*−1/2.
