# Supplementary figures and images for: Cystal structre of 5-hy­droxy-2-nitro­benzaldehyde
Source: Acta Crystallogr E Crystallogr Commun. 2015 Apr 22;71(Pt 5):o328–9. doi: 10.1107/S205698901500701X (PMC4420084; doi:10.1107/S205698901500701X)

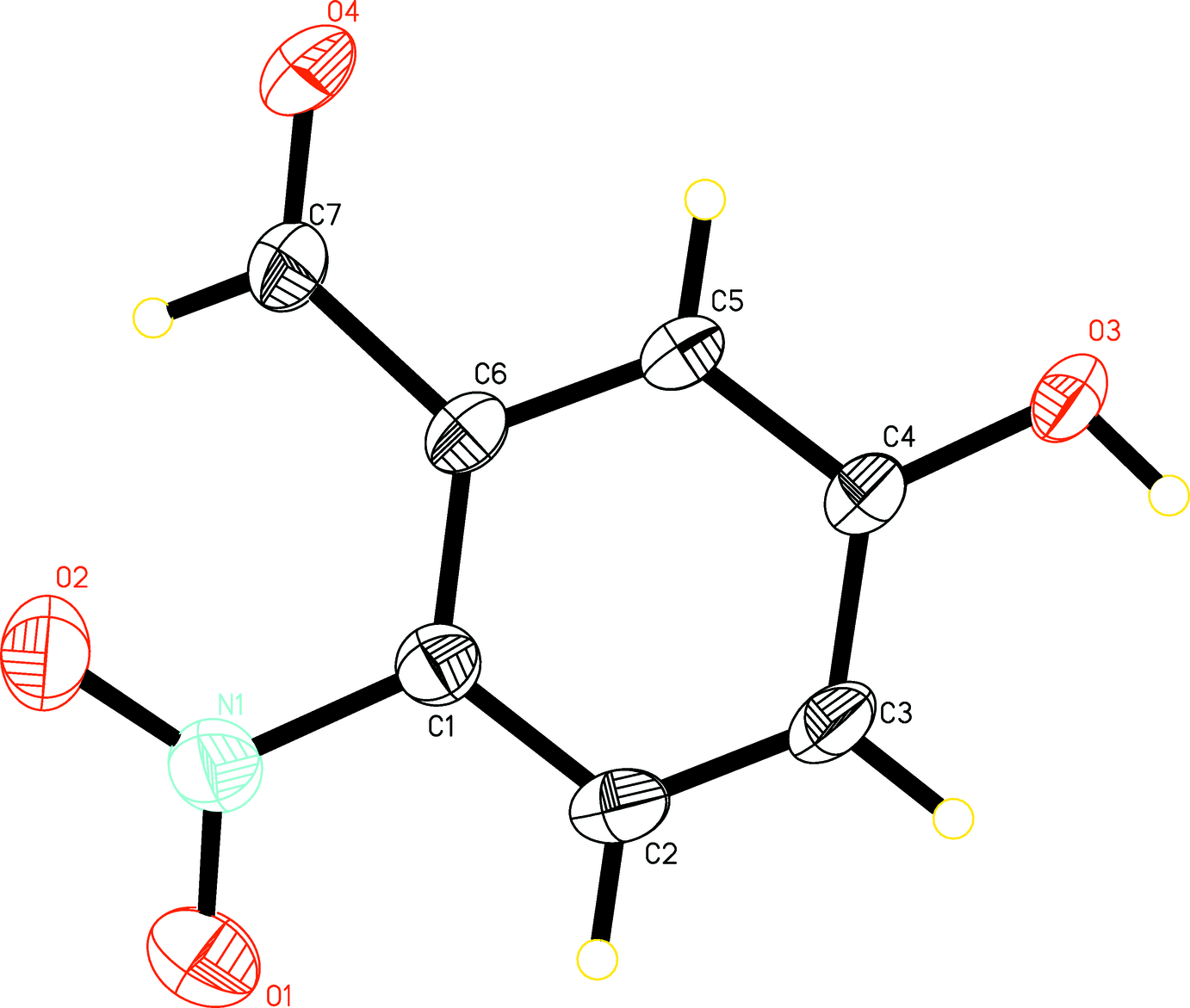

Supplement: Supplementary file 4 [file e-71-0o328-fig1.tif]

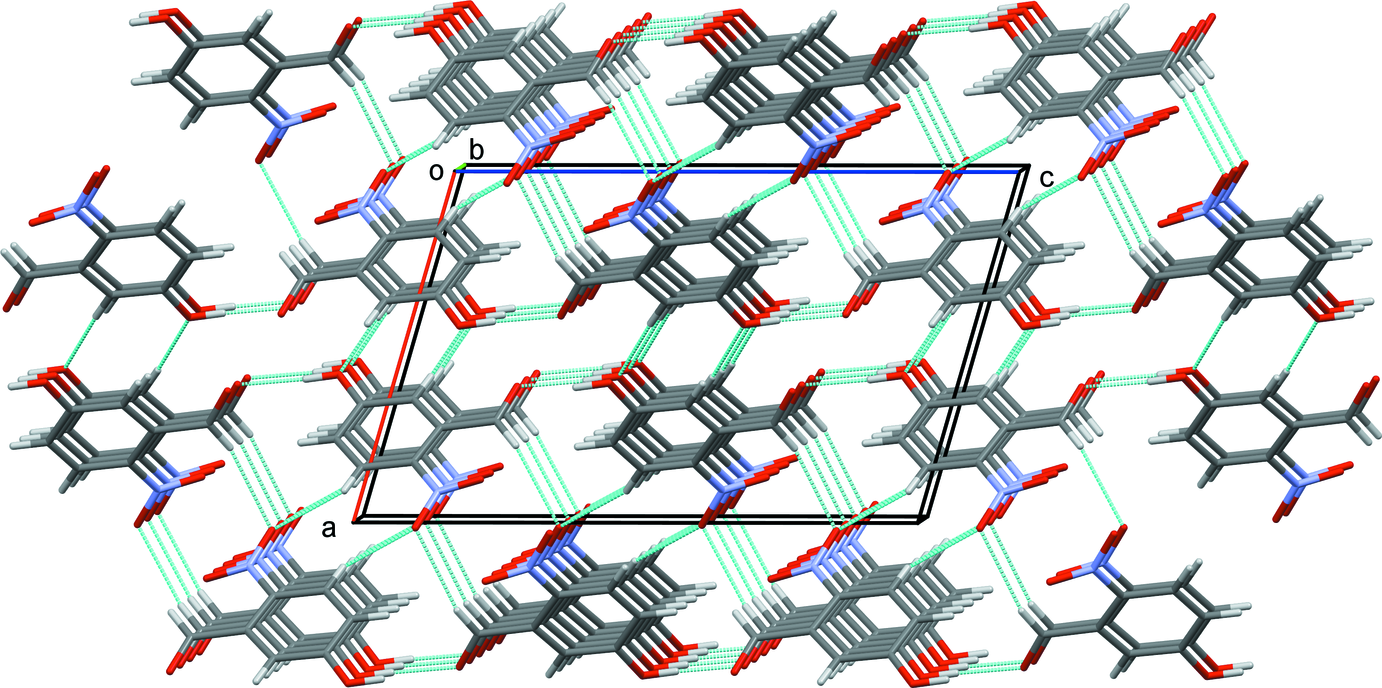

Supplement: Supplementary file 5 [file e-71-0o328-fig2.tif]
